# Emerging Infections: *Shewanella* – A Series of Five Cases

**DOI:** 10.4103/0974-2727.72150

**Published:** 2010

**Authors:** Krishna Kanchan Sharma, Usha Kalawat

**Affiliations:** Department of Microbiology, Sri. Venkateswara Institute of Medical Sciences, Tirupati, Andhra Pradesh, India

**Keywords:** Rare pathogens, non-healing ulcer, chronic ulcer, *Shewanella* algae infection, *Shewanella putrefaciens*, emerging pathogens

## Abstract

**Background:**

*Shewanella* spp. are unusual cause of disease in humans; however, reports of *Shewanella* infections have been increasing. *Shewanella* is a ubiquitous organism that has been isolated from many foods, sewage, and both from fresh and salt water. Earlier it was named as *Pseudomonas putrefaciens* or *Shewanella putrefaciens*. There are several reports describing this organism causing human infections such as cellulitis, abscesses, bacteremia, wound infection, etc. It is oxidase and catalase-positive non-fermenter gram-negative rod that produces hydrogen sulfide.

**Aims:**

The study was conducted to identify *Shewanella* spp., which was wrongly reported as *Pseudomonas* spp.

**Materials and Methods:**

Clinical samples were cultured as per standard clinical laboratory procedure. We tested the non-lactose-fermenting colonies for oxidase positivity. Oxidase-positive colony was inoculated in triple sugar iron slant (TSI) to know the hydrogen sulfide production. Hydrogen sulfide positive colonies were further tested for citrate, urease, indole, and amino acid decarboxylation and acid and gas production from sugars.

**Results:**

Five isolates identified as *Pseudomonas* spp. during preliminary testing were proved to be *Shewanella* spp. on further testing.

**Conclusions:**

It will help in better understanding the epidemiology, pathogenesis and risk factors associated with these and prevention of the rare pathogenic organisms.

## INTRODUCTION

*Shewanella* spp. is a saprophytic gram-negative rod, belonging to family Vibrionaceae. It is frequently isolated from nonhuman sources and is rarely considered pathogenic in humans. *Shewanella* spp. are widely distributed in nature, with soil and water being their natural habitat. Initially they were considered to be colonizers or saprophytes thriving on previously damaged tissue.[1] There are several reports of isolation of *Shewanella* algae from various clinical samples. *Shewanella putrefaciens*, which was first described and named by Mac Donell and Colwell in 1985, is only known non-fermentative gram-negative rod that produces hydrogen sulfide.[2] It was first isolated from tainted butter by Derby and Hammer in 1931 and was classified as *Achromobacter putrefaciens*.[3]

*Shewanella putrefaciens* has only been isolated from such clinical materials as various body wounds, feces, conjunctiva, urine, CSF, bile, ascitic fluid, pleural fluid, and stored blood. The major risk factor of *S. putrefaciens* infection is hepatobiliary disease, peripheral vascular disease, with chronic leg ulcer, poor hygiene, and socioeconomic status. In most cases, the bacteria reside in devitalized tissue or denuded skin and serve as a nidus for opportunistic infection. Soft tissue infections have various clinical manifestations including infected leg ulcer, cellulitis, abscess formation, and wound infection, which are often preceded by chronic ulceration of the lower limb, trauma, burn wound, and seawater exposure.[4–7] Often in clinical laboratory oxidase-positive non-fermenter gram-negative rods grown on routine laboratory culture media are considered as *Pseudomonas* spp. and no further identification is made. Therefore, so called *Pseudomonas* should be further studied to exclude other oxidase-positive gram-negative rods that are thought to be rare. *Shewanella* spp. are very easy to identify and can be easily grown on routine bacteriological media. Thus we tried to find out as to how many of *Shewanella* spp. are wrongly reported as *Pseudomonas* spp. by just doing simple biochemical tests over a period of six months. Here we present a series of five cases identified as *Shewanella* infection during the six months study period.

## MATERIALS AND METHODS

Clinical samples were cultured on blood agar, MacConkey agar, chocolate agar and *Salmonella–Shigella* agar for stool sample. Culture plates were incubated at 37°C for 18–24 h. If there was no growth after overnight incubation, plates were further incubated overnight. Bacterial colony on blood agar and MacConkey agar was tested for oxidase production by strip impregnated with 1% tetramethyl-p-phenylenediamine-dihydrochloride. If colony gave blue color within 30 seconds, it was considered as oxidase-positive organism. Oxidase-positive colonies were further characterized by gram stain, urease on Christensen’s urea agar, citrate utilization by Simmons citrate agar, H_2_S production in sulfide indole motility (SIM) and TSI slant for differentiation between fermenter and non-fermenter and H_2_S production. Oxidase-positive non-fermenter gram-negative bacilli producing H_2_S gas in TSI slant were identified as *Shewanella* spp. since this is the only non-fermenter which produces hydrogen sulfide gas in TSI agar and is also oxidase-positive.[1]

*Shewanella* spp. were further identified and differentiated into *S. algae* and *S. putrefaciens* based on, nitrate reduction, metabolism of maltose, mannitol, sucrose, arabinose, ribose, decarboxylation of amino acids arginine, lysine, and ornithine, hemolysis on 5% sheep blood agar, growth on *Salmonella–Shigella* (SS) agar, growth at 42°C, and growth in presence of NaCl 6.5% w/v. Antibiotic sensitivity testing was done using Kirby–Bauer disc diffusion method following NCCLS guidelines with ampicillin, amikacin, ceftazidime, piperacillin, piperacillin/tazobactam, tobramycin, cefoperazone/sulbactam, ciprofloxacin, polymyxin B, and zone of inhibition was measured and reported as sensitive or resistant.[8] The results are depicted in Tables 1 and 2. Blood hemogram was done on automation five-part differential cell counter, and biochemical analysis of blood was performed on Beckman auto-analyzer Synchron CX-9.

**Table 1:** Various biochemical reactions to oxidase-positive non-fermenting gram-negative bacilli producing hydrogen sulfide gas

Biochemical test ↓	Isolate from case 1	Isolate from case 2	Isolate from case 3	Isolate from case 4	Isolate from case 5
Catalase	P	P	P	P	P
Oxidase	P	P	P	P	P
Citrate	N	N	N	N	N
Urease	N	N	N	N	N
Indol	N	N	N	N	N
H_2_S in TSI	P	P	P	P	P
Ornithine decarboxylase test	P	P	P	P	P
Arginine decarboxylase test	N	N	N	N	N
Lysine decarboxylase test	N	N	N	N	N
Growth on 6.5% NaCl Agar	P	P	P	P	P
Hemolysis on sheep blood agar	P	P	N	P	N
Acid from mannitol	N	N	N	N	N
Acid from ribose	P	P	N	P	N
Acid from maltose	N	N	P	N	P
Acid from sucrose	N	N	N	N	N
Acid from arabinose	P	P	N	P	N
Growth on SS agar	P	P	N	P	N
Growth at 42°C	P	P	N	P	N
Interpretation	*S. algae*	*S. algae*	*S. putrefaciens*	*S. algae*	*S. putrefaciens*

TSI – triple sugar iron, SS agar - Salmonella-Shigella agar, P - positive, N - negative

**Table 2:** Antibiotic sensitivity of isolates

Case no.	A	Ak	Ca	PIP	PT	Tobra	CFS	CF	PB
Case 1	R	S	S	S	S	S	S	R	S
Case 2	R	S	S	R	S	S	S	R	S
Case 3	R	S	R	S	S	S	S	R	S
Case 4	R	S	S	S	S	S	S	R	S
Case 5	R	S	S	S	S	S	S	S	S

A - ampicillin, Ak - amikacin, Ca - ceftazidime, PIP - piperacillin, PT - piperacillin/tazobactam, Tobra - tobramycin, CFS - cefoperazone/sulbactam, Cf - ciprofloxacin, PB - polymyxin B, S - sensitive, R - resistant

### Case 1

A 43-year-old male with a history of non-healing traumatic ulcer of left foot for the last seven years came to the plastic surgery out patients department (OPD). Patient had a history of injury to the left foot by run over of the lorry tyre seven years back. Superficial grafting was done at a local hospital but graft loss occurred within seven days. Patient was referred to higher center, where free microvascular flap was done. Graft loss occurred after 3 months. Re-grafting was done, which was again followed by loss of graft in three months. Since then he had raw area getting treated with dressings. Patient had no history of tuberculosis, diabetes, hypertension, and any allergy.

Patient was negative for HIV and HBsAg by ELISA. Hemogram was as follows: blood hemoglobin-14.5 g/dL, WBC - 8100/cm, neutrophil - 65%, eosinophil - 3%, lymphocyte - 27%, monocytes - 5%. Biopsy report was as follows: acanthotic keratinized epidermis with subepithelial proliferating capillaries, mononuclear infiltrate, congestion, hemorrhages, hemosiderin-laden macrophages, and no sign of malignancy. Venous Doppler report showed left great saphenous vein dilated and left saphenofemoral function incompetent. Reflux of flow was noted with Valsalva maneuver in the course of great saphenous vein. One perforator was seen just below leg, 3 cm above the ankle. The ulcer healed successfully after one year of conservative management. Wound swab culture showed growth of *Shewanella algae* and coagulase-negative staphylococci.

### Case 2

A 62-year-old male with type 2 diabetes mellitus, had left foot cellulites, multiple ulcers, and great toe amputation. He was diagnosed as a case of peripheral neuropathy. No history of injury was present. His glycosylated hemoglobin (HbA1c) was 5.7%, serum creatinine - 0.8 mg%, triglyceride - 46 mg% and high-density lipoprotein - 35 mg%. Patient had a history of generalized body itching with burning and numbness of the foot. There was swelling and discharge from the lesion at the left foot. There was no fever, chest pain, palpitation, no abdomen pain, or diarrhea. On examination, there was pallor and dehydration, pulse 96/min, blood pressure 110/70 mm of mercury, cardiovascular and respiratory systems were normal, and abdomen was soft and no abnormality was detected. On local examination of the foot swelling, local rise of temperature, discharge, and interdigital ulceration was observed.

### Case 3

A 57-year-old female, a known case of carcinoma breast, operated 16 years back, developed non-healing ulcer on chest wall in the left axillary region. She complained of pain in the chest wall. The ulcer measured 5×3 cm and was fixed to the chest wall. Surrounding skin was indurated. Mammogram did not show any focal lesion. Punch biopsy did not show any evidence of malignancy. She was hepatitis B positive and all other systemic parameters were normal. Debridement was done and heterograft applied. The ulcer healed over a period of approximately six months with rigorous treatment.

### Case 4

A 34-years-old male, farmer by occupation was suffering from severe gastroenteritis with complaints of diarrhea and vomiting for five days, and dyspnea, oliguria, and hematemesis for two days. There was no history of diabetes, hypertension, smoking, alcohol intake, or any other chronic illness or prolonged hospitalization. On examination, blood pressure was 190/110 mm of mercury, pulse rate 120/minute, rapid and feeble in character. There was no fever and no signs of cardiac failure. His blood parameters were as follows: hemoglobin 15.2 g/dL, hematocrit 42.3%, red blood cell count 5.58 × 10^6^ mm^3^, platelet count 2.16 × 10^5^/mm^3^, total WBC count 28300/mm^3^, ESR 6 mm fall in first hour by Westergren method, random blood sugar 170 mg/dL, urea 316 mg/dl, creatinine 20.14 mg/dL, conjugated bilirubin 1.1 mg/dL, unconjugated bilirubin 0.3 mg/dL, aspartate aminotransferase and alanine aminotransferase were 39, 36 U/L, respectively. Biochemical parameters gave the evidence of renal failure. Patient was treated with diuretics and intravenous sodium bicarbonate. He was also given calcium channel blocker - amlodipine 5 mg, ciprofloxacin 200 mg, and metronidazole 500 mg parentally twice a day, to cover up blood pressure and aerobic and anaerobic bacterial infection. After microbiological report, patient was shifted from ciprofloxacin to ceftriaxone along with metronidazole. Five sessions of hemodialysis were given. Diarrhea was controlled but renal and respiratory parameters worsened. He developed metabolic acidosis, pulmonary edema and then had sudden cardiac arrest and succumbed. Stool was examined for presence of ova, cyst, and trophozoites by saline and iodine preparation.[9] Stool culture was performed on SS agar and MacConkey agar. Blood culture was sterile. Stool culture showed growth of *Shewanella algae* and *E. coli*.

### Case 5

A 35-year-old female suffered from burns, reported with raw area on right leg extending from lower 1/3^rd^ of leg to the dorsum of foot. All her biochemical parameters were within normal limits and all vital signs were normal. The raw area was debrided and heterograft applied. She responded well and the burn area healed completely.

## RESULTS

There was mixed growth in Cases 1 and 4. *Shewanella algae* were grown along with coagulase-negative Staphylococci in patient 1 and *E. coli* in patient 4. In other cases, it was pure growth of *Shewanella* spp. Isolates were identified as *Shewanella algae* in Cases 1, 2, and 4 and *S. putrefaciens* in Cases 3 and 5.

## DISCUSSION

*Shewanella* spp. have been associated with several kinds of infections like biliary tract infection, empyema, skin, and soft tissue infections such as fulminant periorbito-facial cellulitis, dacrycystitis, perianal abscess, finger abscess, traumatic lesions or burns of the lower limbs, bacteremia, and rheumatic heart disease. It has also been reported in premature babies with pneumonia.[10] Most of these patients had predisposing factors such as malignancy, hepatobiliary disease, neutropenia, or prematurity.

The first description of the species was provided in 1931 by Derby and Hammer.[3] Initially, *Shewanella algae* was misidentified as *S. putrefaciens* by most of the investigators; it was as late as 1980, when Gillardy,[11] recognized three biovars and CDC recognized two biotypes based on carbohydrate oxidation and growth on SS agar and nutrient agar containing high salt concentration (6.5%). Prior to the identification of *S. algae*, most of the human infections caused by *Shewanella* spp. were believed to be caused by *S. putrefaciens*, which was first reported in clinical isolates by King.[12] Simudu proposed the name of *Shewanella algae*.[13]

Later on, several other phenotypic and genotypic characteristic were described to differentiate between the two biotypes. Recent results of 16S rRNA gene sequence analyses of genera from this group led to a proposal for a new family called Shewanellaceae, containing about 30 *Shewanella* spp., most of which are psychrophilic and therefore of little interest to clinical microbiologists.[11] The only *Shewanella* spp. found in clinical specimens are *S. putrefaciens* and *S. algae*.

Most of the human infections are reported to be caused by *S. algae*.[14–16] Mouse pathogenicity study performed by Khashe and Janda indicated that *S. algae* were the more virulent species, and the hemolytic activity of *S. algae* was speculated to be an important virulence factor.[15] Automated identification systems are unable to distinguish between *S. putrefaciens* and *S. algae*. A large number of clinical isolates identified tentatively as *S. putrefaciens* were shown to be *S. algae* by Nozue *et al*.[14] A number of reports of human infections allegedly caused by *S. putrefaciens* do not supply sufficient data to show whether or not the isolates were actually *S. algae*.[17]

In the present study also, *S. algae* was isolated in three of the five cases and *S. putrefactions* only in two. Most common infections due to *Shewanella* spp. are infections of skin and soft tissue, and are usually associated with breaches in the skin such as ulcers or following trauma.[18,19] Among our isolates, four were from pus samples from skin infections and one from stool.

The antibiotic sensitivity was determined by Kirby–Bauer disk diffusion method, which was similar to that published earlier.[19] According to the literature, most *Shewanella* infections are treated easily by a combination of surgical therapy, drainage and antibiotics.[20,21] Poor outcome is associated most often with underlying disease.[22] Treatment options include β-lactamase antibiotics provided that the strain is susceptible. All our cases responded well to treatment except for the gastrointestinal infection patient where there was mortality. The patient succumbed to the renal failure and its complications, though the isolate was sensitive to most of the conventional antibiotics. In this case it was lactose and sorbitol fermenting *E. coli*, thus renal failure or hemolytic uremic syndrome could be due to enteropathogenic *E. coli* as well.

In this study, four of the five cases were skin and soft tissue infections. We did not find any systemic inflammatory response in any of the soft tissue infection cases. All soft tissue infections having chronic ulceration of prolong duration which supports the theory that there is often a coexistence of defects in both nonspecific and specific host defense mechanisms may explain why the patients did not mount a remarkable systemic inflammatory response.[4–6]

Though soft tissue and wound infection due to *Shewanella* spp. have been reported from several countries, such reports from India are rare. The main reason for this is that in most of the hospitals, all gram-negative and oxidase-positive organisms are reported as *Pseudomonas* spp. and no further identification is done.

So there is need to look for such rare organisms and not to dispose all oxidase-positive organisms as *Pseudomonas*. It may not affect the overall outcome of the patient, but it will definitely help in better understanding of the epidemiology, pathogenesis, and preventive aspects of such organisms.
